# Two Catastrophes in One Patient: Drug Reaction with Eosinophilia and Systemic Symptoms and Toxic Shock Syndrome

**DOI:** 10.7759/cureus.1359

**Published:** 2017-06-15

**Authors:** Moayed Ibrahim, Diana L Nunley

**Affiliations:** 1 Internal Medicine, Quillen College of Medicine at East Tennessee State University

**Keywords:** tss, dress, immunodeficiency, clopidogrel

## Abstract

A 70-year-old, immunocompromised patient presented to the emergency room (ER) five weeks after she was started on clopidogrel. She complained of skin eruption, mouth ulcers, fatigue, and myalgia over the past two weeks. Labs showed severe hyponatremia, acute kidney injury, rhabdomyolysis, hyperkalemia, and elevated liver enzymes. She was treated with steroids and discharged after her condition improved. However, a month later, she returned to the ER, complaining of nausea, vomiting, diarrhea, dizziness, chills, and shortness of breath over the past two days. She was lethargic and had orthostatic hypotension. She deteriorated clinically within a few days, with worsening lethargy and the development of respiratory distress along with profound hypotension. She needed mechanical ventilation and vasopressors. In addition, she had melena, severe thrombocytopenia, and hemolytic anemia. With supportive care, she improved and was discharged after a long stay in the intensive care unit. Retrospectively, the first hospitalization was believed to be caused by drug reaction with eosinophilia and systemic symptoms (DRESS). Treating that with steroids compromised her immune system beyond her pre-existing primary immunodeficiency status. At the time of her second hospitalization, she met the Centers for Disease Control and Prevention (CDC) criteria for a toxic shock syndrome (TSS) diagnosis. Her TSS started four days after a skin biopsy, which was done as part of her skin rash workup. It was thought that the source of the exotoxin that mediated her TSS was her skin, given the temporal relationship of the skin biopsy to her TSS. Another potential source of the exotoxin was the gastrointestinal tract, given the predominant gastrointestinal symptoms she had at the time of her second admission.

## Introduction

Drug reaction with eosinophilia and systemic symptoms (DRESS) is a rare, potentially life-threatening, drug-induced hypersensitivity reaction that includes skin eruptions, hematologic abnormalities (eosinophilia and atypical lymphocytosis), lymphadenopathy, and internal organ involvement (liver, kidney, and lungs) [1-3]. Reported mortality rates are between 5% and 10% [4-5]. Toxic shock syndrome (TSS) is another, rare clinical entity, which is a potentially fatal illness caused by a bacterial toxin. It typically presents in patients with streptococcal or staphylococcal skin infections. The diagnosis of DRESS and TSS remains challenging and requires a high index of suspicion. The most common offending drugs that cause DRESS are sulfonamides and antiepileptic medications. However, our patient in this report developed DRESS in reaction to clopidogrel. In addition, she developed TSS while on steroids for DRESS. To our knowledge, this is the first case report in medical literature describing DRESS followed by TSS in the same patient.

## Case presentation

A 70-year-old patient with a history of remote endometrial cancer, primary immunodeficiency, recurrent herpes simplex virus 1 infections, hypertension, type 2 diabetes mellitus, and a transient ischemic attack presented to the emergency room (ER) with a pruritic skin rash, painful mouth sores, fatigue, and diffuse severe myalgia for the past two weeks. Five weeks earlier, at her request, she was switched to clopidogrel from aspirin/dipyridamole due to affordability factors. A physical exam revealed an edematous flushed face with red raised papules and blistering oral mucosal lesions. The papules also covered her upper extremities and spread the next day to her torso and thighs. The papules coalesced within a couple of days over the chest and the left proximal thigh, where she also had blisters. There was no heliotrope rash or Gottron's papules. Initial labs revealed severe hyponatremia, acute kidney injury, rhabdomyolysis, hyperkalemia, and elevated liver enzymes. Complete blood counts revealed severe thrombocytopenia and leukocytosis, but no eosinophilia. A peripheral blood smear did not show atypical lymphocytes. Titers of herpes simplex virus 6 (HSV 6) and immunoglobulin G (IgG) were positive but negative for Immunoglobulin M (IgM). Titers of the hepatitis panel, rheumatoid factor, Anti-Smith, Anti-Jo-1, Anti-Mi-2, and antinuclear antibodies were negative as well. At that time, it was thought that this could be a clopidgorel-related systemic and skin reaction. Therefore, clopidogrel was discontinued and the patient was started on a high dosage of steroids with significant improvement in the rash. Eventually, she was discharged with an outpatient dermatology follow-up.

She had a skin biopsy one month from her hospitalization date. The biopsy revealed interface vacuolar degeneration, mild perivascular inflammation, solar elastosis, and dermal mucinosis. These changes were nonspecific but can be seen with DRESS. The fact that she was treated with steroids preceding the biopsy might have altered the histopathology. Unfortunately, four days after the skin biopsy, the patient returned to the ER complaining of nausea, vomiting, diarrhea, dizziness, chills, and shortness of breath for the past two days. On presentation, she had profound hypotension, possibly related to being dehydrated or an underlying sepsis. She was lethargic and had dry mucus membranes. A skin exam revealed a residual rash, but it was significantly improved compared to the first admission. Initial labs revealed mild hyperkalemia and severe hyponatremia. Preliminary cultures of blood, urine, and sputum were unrevealing. Testing was negative for influenza A and B as well as Legionella. Within two days of presentation, her altered mentation worsened; she developed significant respiratory distress and her hypotension persisted. Her chest X-ray and head scan were negative and within normal limits. She was intubated on the fourth hospital day, as she was becoming unresponsive. She required vasopressor support and antibiotics were started for possible sepsis. Furthermore, she had a lower gastrointestinal bleed, severe thrombocytopenia, and hemolytic anemia. Eventually, with continued supportive measures, she came off the vasopressors and ventilation. Intravenous antibiotics were discontinued because no source of infection was found. Her final blood, sputum, and urine cultures were negative as well. Her overall clinical condition, including kidney function and liver enzymes, improved before discharge, unlike her thrombocytopenia and creatinine kinase elevation, which persisted for three months before returning to baseline. She was discharged to physical rehab after a month-long stay at the intensive care unit.

## Discussion

This case narrates a rare, life-threatening adverse reaction to clopidogrel. Clopidogrel is a thienopyridine drug that inhibits platelet aggregation and has many indications in cardiac and neurological vascular diseases. The most common hypersensitivity reactions to clopidogrel are dermal eruptions and severe systemic symptoms, which are reported in less than one percent of case reports. The most common dermal eruptions are DRESS and acute generalized exanthematous pustulosis (AGEP). The latency period of DRESS is two to eight weeks, and it primarily affects the face and chest and then spreads to the abdomen and extremities. In one-third of the cases, it can involve the entire body. It has a prolonged course, can relapse despite discontinuation of the offending drug, and is usually associated with a reactivation of HSV 6. Skin involvement includes purpura, scaling, and facial edema. Typically affected internal organs are the kidneys, brain, nerves, and muscles. On the other hand, the latency period for AGEP is 5 to 10 days and it presents with bullous rash, arthralgia, bronchospasm, liver injury, pancytopenia, kidney injury, and anasarca. In a literature review [6] published in 2011, Cacoub P et al. found that eosinophilia and atypical lymphocytes were found in 66% and 27%, respectively, of the 174 DRESS cases reported between January 1997 and May 2009 in PubMed and MEDLINE. Our patient did not have either.

The sequence of events in our patient was DRESS in reaction to clopidogrel, treatment of that with steroids, coupled with her pre-existing immunodeficiency and type 2 diabetes mellitus, which increased her risk for infections. It was found in a study [7] conducted by Smit J et al. that glucocorticoids can increase the risk of community-acquired Staphylococcus aureus bacteremia. While the blood and sputum cultures were negative for Staphylococcus aureus, TSS is mediated by a superantigen toxin that results from an infection, most commonly that of the skin. In this patient, the source of the exotoxin was either the skin, given the fact that her TSS started after only four days of the skin biopsy, or the gastrointestinal tract, given that her TSS started with striking gastrointestinal symptoms. An assay for exotoxins is not commercially available, which makes the diagnosis of TSS entirely clinical. The differential diagnosis of TSS, which results from a toxic-mediated capillary leak process, includes infections with dermatological manifestations, such as Rocky Mountain spotted fever (RMSF), leptospirosis, measles, influenza, or Legionella. Our patient was negative for Legionella and the influenza A and B antigens and did not have the features suggestive of RMSF, leptospirosis, or measles. CDC criteria for a confirmed case of TSS include a patient with fever ≥38.9°C, hypotension, diffuse erythroderma, desquamation (unless the patient dies before desquamation can occur), and the involvement of at least three organ systems. A probable case is a patient who is missing one of the characteristics of the confirmed case definition. Based on that, our patient was at least a probable case of TSS, given that the desquamation was not clear on exam. However, it was difficult to elucidate desquamation in our patient, given the peripheral cyanosis and skin necrosis that resulted from vasopressor use.

## Conclusions

DRESS is a rare, serious drug reaction that can be fatal. Antiepileptic medications and sulfonamides are the most common inciting agents. More drugs are being reported to the Food and Drug Administration (FDA) as causative agents of DRESS. In this report, we presented a severe case of DRESS induced by clopidogrel, complicated by TSS, mediated by a skin or gastrointestinal exotoxin, in the setting of the patient’s increased susceptibility to infections given her advanced age, primary immunodeficiency status, type 2 diabetes mellitus, and receipt of high doses of steroids.
